# Differences in the inflammatory response and outcome among hospitalized patients during different waves of the COVID-19 pandemic

**DOI:** 10.3389/fimmu.2025.1545181

**Published:** 2025-03-27

**Authors:** Violeta Briciu, Daniel-Corneliu Leucuta, Monica Muntean, Amanda Radulescu, Cristina Cismaru, Adriana Topan, Lucia Herbel, Melinda Horvat, Mirela Flonta, Mihai Calin, Roxana Dobrota, Mihaela Lupse

**Affiliations:** ^1^ Department of Infectious Diseases and Epidemiology, Iuliu Hatieganu University of Medicine and Pharmacy, Cluj-Napoca, Romania; ^2^ The Clinical Hospital of Infectious Diseases, Cluj-Napoca, Romania; ^3^ Department of Medical Informatics and Biostatistics, Iuliu Hatieganu University of Medicine and Pharmacy, Cluj-Napoca, Romania

**Keywords:** COVID-19, pandemic waves, inflammatory biomarkers, systemic inflammatory indexes, disease severity, mortality

## Abstract

**Introduction:**

The aim of this study was to evaluate differences in inflammatory biomarkers and their association with outcomes in hospitalized patients with coronavirus disease 2019 (COVID-19) during four pandemic waves determined by different SARSCoV- 2 variants of concern. We explored if laboratory biomarkers of inflammation adjusted to patients’ comorbidities, age, and vaccination status correlated with severity and mortality.

**Methods:**

A retrospective study on 8,614 consecutive hospitalized COVID-19 patients was conducted in a Romanian hospital on a 3-year interval (February 2020 to May 2023). Data collected included demographics, duration of hospitalization, comorbidities, vaccination status, COVID-19 severity, outcome, and markers of inflammation from the first blood test performed at admittance [C-reactive protein (CRP), fibrinogen, ferritin, lactate dehydrogenase (LDH), procalcitonin (PCT), IL-6, D-dimer, and complete blood count]. Systemic inflammatory indexes like neutrophil-to-lymphocyte ratio (NLR), derived neutrophil-to-lymphocyte ratio (dNLR), lymphocyte-to-monocyte ratio (LMR), platelet-to-lymphocyte ratio (PLR), neutrophil-to-platelet ratio (NPR), Systemic Immune-Inflammation Index (SII), and systemic inflammation response index (SIRI) were calculated.

**Results:**

The Delta wave, characterized by the longest hospitalizations and the highest rates of severe cases and mortality, showed significant elevations in inflammatory biomarkers. CRP, fibrinogen, ferritin, IL-6, D-dimer, and LDH increased in their median values from the Wuhan to Delta wave and decreased in the Omicron wave, except PCT, which increased from the Alpha to Omicron wave. Leukocytes and neutrophils increased in their median values from the Wuhan to Delta wave and decreased in the Omicron wave, while an inverse pattern can be observed for lymphocytes, monocytes, and basophils. The best inflammatory biomarkers for predicting severe/critical COVID-19 were CRP, dNLR, LDH, and NLR (cut-off of 3.41 mg/dL, 3.05, 262 U/L, and 4.5, respectively), while for predicting death outcomes, the best biomarkers were dNLR, NLR, LDH, and NPR (cut-off of 3.6, 4.9, 278 U/L, and 0.02, respectively). For all these biomarkers, the areas under the curve (AUCs) surpassed 0.8. In the multivariate analysis, the highest adjusted OR for death was described for dNLR (8.46), NLR (7.59), LDH (5.99), and NPR (5.5), while increased lymphocytes decreased the highest adjusted OR for death (0.16).

**Conclusion:**

The study, underscoring the dynamic nature of COVID-19, brings a detailed analysis of biomarker trends that could provide valuable information for the early identification of patients at risk for severe outcomes, allowing for timely interventions.

## 1 Introduction

Severe coronavirus disease 2019 (COVID‐19) is a hyperinflammatory syndrome. The severe outcome among patients with SARS-CoV-2 infection is related to the cytokine storm and hyperinflammation, responsible for acute respiratory distress syndrome and multiple organ failure (1). Cytokines are immunomodulating agents that are fundamental mediators for establishing communication among the immune system cells. The body requires a homeostatic balance of cytokine levels, which, if perturbed, could harm the host system. COVID-19-associated cytokine storm patients display elevated levels of several critical proinflammatory cytokines, like interferon-gamma (IFN-γ), tumor necrosis factor-alpha (TNF-α), interleukin 1 (IL-1), interleukin 2 (IL-2), interleukin 6 (IL-6), IFN-γ-inducible protein 10 (IP-10), monocyte chemoattractant protein-1 (MCP-1), granulocyte–macrophage colony-stimulating factor (GM-CSF), and interleukin 10 (IL-10), and the levels of these cytokines were found to correlate with the severity of the disease (2, 3).

However, inflammatory biomarkers such as C-reactive protein (CRP), IL‐6, ferritin, D‐dimer, and procalcitonin (PCT) are commonly measured in clinical practice and have been used in attempts to estimate the prognosis of patients with COVID‐19 (4, 5) and guide immunomodulatory therapy (6, 7). CRP, ferritin, D-dimer, white blood cells, and IL-6 have been identified by the WHO as key biochemical parameters for the management of COVID-19 patients (8). It still remains a challenge to determine which of the hyperinflammatory biomarkers and cytokines are the best predictors of disease severity and mortality in COVID-19 patients.

The complement system, a key player in the innate immune response, has also been implicated in the pathogenesis of severe COVID-19, as complement activation is a key mediator of thrombosis, inflammation, and tissue damage during acute SARS-CoV-2 infection (9). The system can be activated by three arms—the classical, lectin, or alternative pathway—and increased complement activation proved to be an immunological feature of COVID-19, which distinguishes those developing severe illness (10). Multiple studies have demonstrated elevation of C5a and soluble C5b-9 in patients with COVID-19 (11), as well as the deposition of activated complement proteins in injured tissues and organs (12), thus creating a precedent for targeting the complement system in clinical trials using complement inhibitors in COVID-19 (13).

The SARS-CoV-2 virus has suffered genetic variation since the onset of the pandemic. Phenotypic impacts of the different circulating variants of concern (VOCs) of the SARS-COV 2 virus (like impact on transmissibility, disease severity, risk of reinfection, and impact on diagnosis) have been monitored by the WHO based on available scientific data throughout the pandemic. Disease severity and risk of hospitalization increased as new VOCs appeared, except for the Omicron VOC, which was characterized by reduced risk of hospitalization and disease severity compared with the previous Delta VOC (14). Although the exact mechanisms behind these disparities are still being researched, the inflammatory response and clinical manifestations of COVID-19 may also differ among these variants. The improved prognosis of the Omicron wave may be related, in addition to extended protection by vaccination/previous infection or better access to medication, to the virus’s reduced capacity to provoke a systemic inflammatory response. There are only a few published studies that have evaluated the inflammatory biomarkers in relation to the genetics of the virus throughout the pandemic (15–17) and even fewer on a large number of patients. A large international study on 3,099 COVID-19 patients from the USA and Europe evaluated the inflammatory biomarkers by comparing the Omicron to pre-omicron variant status (18).

Therefore, we aimed to perform research on a larger number of patients and to explore to a greater degree the comparisons between different pandemic waves. The aim of this study was to assess the relationship between inflammatory biomarkers and outcomes in hospitalized COVID-19 patients across four pandemic waves, defined by the Wuhan strain and the Alpha, Delta, and Omicron variants of concern.

## 2 Materials

### 2.1 Study design and setting

A retrospective study on consecutive hospitalized patients was conducted in the Clinical Hospital of Infectious Diseases in Cluj-Napoca, Romania, beginning with the first COVID-19 hospitalized case (27 February 2020) and ending on 31 March 2023, the last month of the pandemic as declared by the WHO (19).

### 2.2 Participants

Inclusion criteria were as follows: hospitalization for COVID-19 diagnosis (positive SARS-CoV-2 molecular diagnostic or rapid antigen test) and age ≥ 18 years.

### 2.3 Variables and measurements

Data collected included age, gender, comorbidities, vaccination status, admittance date, duration of hospitalization, intensive care unit (ICU) admission, and clinical outcome. The severity of COVID-19 was defined as asymptomatic, mild (without pneumonia), medium (with non-severe pneumonia), and severe/critical (severe: tachypnea with >30 breaths/min or oxygen saturation <93% at rest or PaO_2_/FIO_2_ < 300 mmHg; critical: respiratory failure requiring invasive or non-invasive mechanical ventilation, shock, or other organ failure that requires intensive care), according to the first World Health Organization classification (20) and adopted by a Romania Health Ministry Order on COVID-19 management. Disease severity was established at the end of hospitalization.

The markers of inflammation of the first blood test performed at admittance were also collected: CRP, fibrinogen, ferritin, lactate dehydrogenase (LDH), PCT, IL-6, D-dimer, and complete blood count. The following indexes were calculated: neutrophil-to-lymphocyte ratio (NLR); derived neutrophil-to-lymphocyte ratio (dNLR), calculated as the ratio of neutrophils to leukocytes − neutrophils; lymphocyte-to-monocyte ratio (LMR); platelet-to-lymphocyte ratio (PLR); neutrophil-to-platelet ratio (NPR); Systemic Immune-Inflammation Index (SII) (neutrophils × platelets/lymphocytes); and systemic inflammation response index (SIRI) (neutrophil count × monocyte count/lymphocyte count).

The study interval was divided based on national data on the circulation of SARS-CoV-2 VOCs (21): the first wave (ancestral Wuhan strain), which was the period of 27 February 2020 to Week 6 of 2021; the second wave (Alpha VOC), which was the period Week 7 of 2021 to Week 28 of 2021; the third wave (Delta VOC), which was the period Week 29 of 2021 to Week 52 of 2021; and the fourth wave (Omicron VOC) (Week 1 of 2022 to 31 March 2023).

The study was conducted in accordance with the Declaration of Helsinki and approved by the Ethics Committee of the Clinical Hospital of Infectious Diseases in Cluj-Napoca (5824/03.04.2024).

### 2.4 Statistical analysis

All qualitative variables were summarized as counts (n) and percentages (%), while quantitative variables were described using medians and interquartile ranges (IQRs). Comparisons between COVID-19 waves (Alpha, Delta, Omicron, and Wuhan) utilized the chi-square test or Fisher’s exact test for categorical variables and the Kruskal–Wallis test for continuous variables, with non-parametric *post-hoc* pairwise comparisons. Classification between severe/critical and mild cases, as well as between death outcomes and survival, employed receiver operating characteristic (ROC) curves, determining optimal cut-offs by maximizing Youden’s index and computing the corresponding sensitivity (Se) and specificity (Sp). Multivariate logistic regression models, adjusted for age (≥65 years), sex, COVID-19 wave, obesity, diabetes, cardiovascular, pulmonary, hepatic, renal, neurologic diseases, cancers, and vaccination status, were used to analyze inflammatory biomarkers. Assumptions for the models were verified, including checks for multicollinearity and goodness of fit. For all statistical tests, the level of significance was 0.05, and the two-tailed p-value was computed. All statistical analyses were carried out in the R environment for statistical computing and graphics (R Foundation for Statistical Computing, Vienna, Austria), version 4.3.2 (22).

## 3 Results

**Figure 1:**
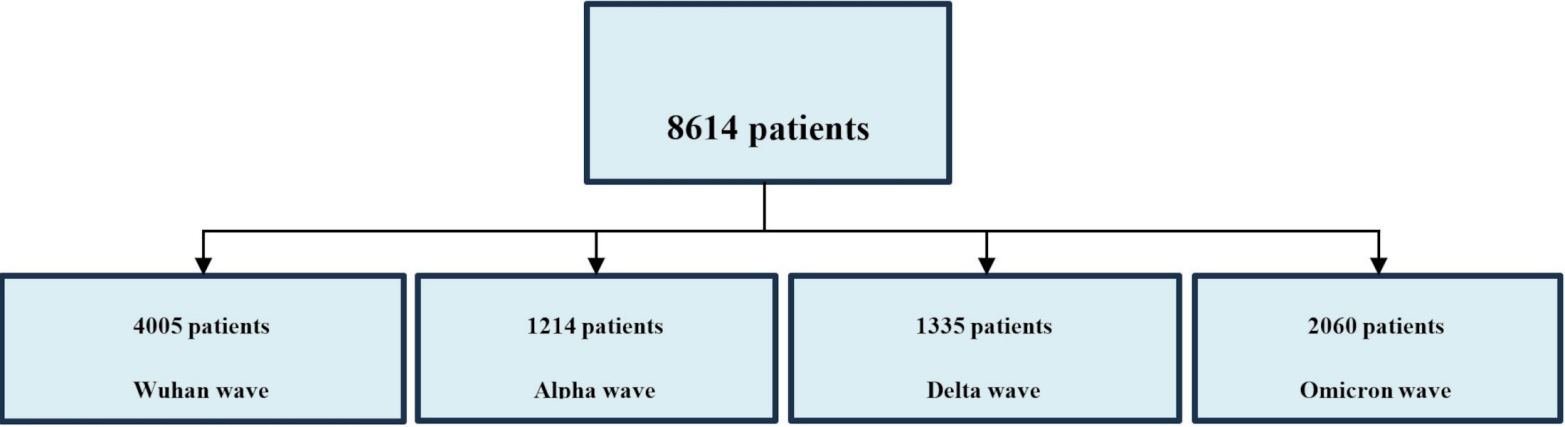
Distribution of study patients and time intervals associated to different pandemic waves in Romania.

A total of 8,614 patients were included in the study, and the time interval and associated waves are presented in **Figure 1**. Patients’ demographics, comorbidities, duration of hospitalization, COVID-19 severity forms, and outcomes during the four waves are presented in **Table 1**.

**Table 1:** Patients' characteristics by wave.

Wave	Wuhan (n = 4,005)	Alpha (n = 1,214)	Delta (n = 1,335)	Omicron (n = 2,060)	p-Value
Age (years), median (IQR)	54 (40–67)	63 (50–72)	64 (51–74)	70 (49–80)	<0.001
Age ≥ 65 years, n (%)	1,193 (29.79)	547 (45.06)	662 (49.59)	1,275 (61.89)	<0.001
Sex (F), n (%)	2,119 (52.91)	603 (49.67)	737 (55.21)	1,230 (59.71)	<0.001
Cardiovascular, n (%)	1,720 (42.95)	721 (59.39)	853 (63.9)	1,199 (58.2)	<0.001
Diabetes, n (%)	634 (15.83)	270 (22.24)	324 (24.27)	413 (20.05)	<0.001
Obesity, n (%)	843 (21.05)	541 (44.56)	460 (34.46)	383 (18.59)	<0.001
Endocrine, n (%)	205 (5.12)	91 (7.5)	112 (8.39)	120 (5.83)	<0.001
Pulmonary, n (%)	254 (6.34)	122 (10.05)	184 (13.78)	282 (13.69)	<0.001
Neurologic, n (%)	246 (6.14)	113 (9.31)	161 (12.06)	344 (16.7)	<0.001
Hepatic, n (%)	148 (3.7)	49 (4.04)	85 (6.37)	129 (6.26)	<0.001
Renal, n (%)	149 (3.72)	53 (4.37)	96 (7.19)	160 (7.77)	<0.001
Rheumatological, n (%)	43 (1.07)	39 (3.21)	51 (3.82)	74 (3.59)	<0.001
Cancer, n (%)	215 (5.37)	65 (5.35)	100 (7.49)	239 (11.6)	<0.001
Hospitalization time (days), median (IQR)	7 (4–12)	8 (5–12.75)	10 (6–16)	7 (4–12)	<0.001
Severity, n (%)					<0.001
Asymptomatic	472 (11.79)	6 (0.49)	1 (0.07)	16 (0.78)	
Mild	953 (23.8)	99 (8.15)	116 (8.69)	560 (27.18)	
Moderate	1,930 (48.19)	605 (49.84)	361 (27.04)	786 (38.16)	
Severe	519 (12.96)	423 (34.84)	400 (29.96)	443 (21.5)	
Critical	131 (3.27)	81 (6.67)	457 (34.23)	255 (12.38)	
Mild or moderate	2,883 (71.99)	704 (57.99)	477 (36.09)	1,346 (65.34)	
Severe or critical	650 (16.23)	504 (41.52)	857 (64.19)	698 (33.88)	<0.001
Died, n (%)	153 (3.82)	105 (8.65)	178 (13.33)	136 (6.6)	<0.001
Vaccinated, n (%)	1 (0.02)	12 (0.99)	283 (21.2)	1,100 (53.4)	<0.001

IQR, interquartile range; n, number.

A significant increase in patients’ age is described with each new wave, while more female than male patients were hospitalized during each wave except the Alpha wave.

Comorbidities generally increased in percentage from the Wuhan to Delta wave and decreased during the Omicron wave in comparison with the Delta wave. The longest duration of hospitalization was associated with the Delta wave, with similar medians and IQR during the Wuhan and Omicron waves. The highest percentage of severe or critical COVID-19 hospitalized patients and deaths were associated with the Delta wave. Similar to the comorbidity patterns, the severity and deaths increased in percentage from the Wuhan to Delta wave and decreased during the Omicron wave.

**Table 2** presents the median values of inflammatory biomarkers and indexes in relation to SARS-CoV-2 waves, and significant statistical differences in their values between waves.

**Table 2:** Inflammatory markers in function of SARS-CoV-2 waves.

Wave	Wuhan (n = 4,005)	Alpha (n = 1,214)	Delta (n = 1,335)	Omicron (n = 2,060)	p {Alpha–Delta, Alpha–Omicron, Delta–Omicron, Alpha–Wuhan, Delta–Wuhan, Omicron–Wuhan}
CRP (mg/dL)	1.31 (0.24–5.64)	5.86 (2.18–11.36)	6.22 (2.08–13.06)	2.46 (0.74–7.18)	<0.001 {0.326, <0.001, <0.001, <0.001, <0.001, <0.001}
Fibrinogen (mg/dL)	409.7 (311.14–526.68)	494.74 (399.19–610.48)	517.34 (410.24–645.1)	394.53 (321.95–490.08)	<0.001 {0.005, <0.001, <0.001, <0.001, <0.001, 0.017}
IL-6 (pg/mL)	12.7 (3.59–35.16)	17.48 (6.02–41.05)	19.08 (7.65–47.31)	14.74 (5.5–42.09)	<0.001 {0.005, 0.418, <0.001, <0.001, <0.001, <0.001}
PCT (ng/mL)	0.08 (0.05–0.27)	0.08 (0.05–0.23)	0.12 (0.05–0.41)	0.17 (0.05–0.69)	<0.001 {0.068, <0.001, 0.002, 0.626, 0.005, <0.001}
Ferritin (ng/mL)	216.1 (85.5–490.5)	385.1 (182.1–752.7)	399.55 (181.15–807.43)	169.35 (68.12–370.2)	<0.001 {0.848, <0.001, <0.001, <0.001, <0.001, <0.001}
D-dimer (mg/L)	0.47 (0.29–0.95)	0.6 (0.37–1.1)	0.61 (0.38–1.11)	0.58 (0.31–1.24)	<0.001 {1, 0.109, 0.101, <0.001, <0.001, <0.001}
LDH (U/L)	211 (169–286)	303 (228–404.25)	306 (220–430)	200 (166–257)	<0.001 {0.793, <0.001, <0.001, <0.001, <0.001, <0.001}
Leukocytes (10^3^/μL)	6.1 (4.7–8)	6.19 (4.6–8.8)	6.34 (4.6–9)	6 (4.42–8.33)	<0.001 {0.521, 0.094, 0.018, 0.256, 0.042, 0.484}
Neutrophils (10^3^/μL)	3.79 (2.58–5.59)	4.53 (3.03–7.08)	4.82 (3.12–7.39)	4 (2.58–6.22)	<0.001 {0.195, <0.001, <0.001, <0.001, <0.001, 0.007}
Lymphocytes (10^3^/μL)	1.44 (0.97–1.99)	0.96 (0.66–1.42)	0.86 (0.62–1.27)	1.13 (0.73–1.65)	<0.001 {<0.001, <0.001, <0.001, <0.001, <0.001, <0.001}
Monocytes (10^3^/μL)	0.52 (0.39–0.67)	0.41 (0.27–0.6)	0.39 (0.24–0.59)	0.52 (0.36–0.73)	<0.001 {0.162, <0.001, <0.001, <0.001, <0.001, 0.899}
Basophils 10^3^/μL	0.02 (0.01–0.03)	0.01 (0.01–0.02)	0.01 (0.01–0.02)	0.02 (0.01–0.03)	<0.001 {1, <0.001, <0.001, <0.001, <0.001, 0.018}
Eosinophils (10^3^/μL)	0.14 (0.14–0.14)	0.14 (0.14–0.14)	0.14 (0.14–0.14)	0.12 (0.02–0.14)	<0.001 {0.252, <0.001, <0.001, 1, 0.251, <0.001}
Platelets (10^3^/μL)	222 (176–282)	208 (159–285.75)	212 (162–281.5)	201 (157–252)	<0.001 {0.867, <0.001, <0.001, <0.001, <0.001, <0.001}
NLR	2.53 (1.52–4.69)	4.83 (2.62–8.99)	5.52 (2.95–9.92)	3.47 (1.77–7.19)	<0.001 {0.01, <0.001, <0.001, <0.001, <0.001, <0.001}
dNLR	1.77 (1.13–3.08)	3.2 (1.87–5.76)	3.7 (2.07–6.38)	2.18 (1.23–4.19)	<0.001 {0.023, <0.001, <0.001, <0.001, <0.001, <0.001}
LMR	2.9 (1.95–4)	2.47 (1.63–3.66)	2.33 (1.54–3.55)	2.22 (1.39–3.5)	<0.001 {0.102, <0.001, 0.056, <0.001, <0.001, <0.001}
PLR	152.13 (109.39–236.09)	218.77 (144.12–345.62)	244.59 (156.01–370.23)	174.69 (118.83–271.59)	<0.001 {0.002, <0.001, <0.001, <0.001, <0.001, <0.001}
NPR	0.02 (0.01–0.02)	0.02 (0.02–0.03)	0.02 (0.02–0.03)	0.02 (0.01–0.03)	<0.001 {0.102, <0.001, <0.001, <0.001, <0.001, <0.001}
SII	565.33 (310.74–1,171.04)	1,047.28 (495.5–2,197.15)	1,171.16 (527.72–2,551.31)	675.4 (341.52–1,450.93)	<0.001 {0.039, <0.001, <0.001, <0.001, <0.001, <0.001}
SIRI	1.28 (0.71–2.46)	1.8 (0.94–3.79)	2.04 (0.97–4.13)	1.79 (0.85–3.94)	<0.001 {0.152, 0.21, 0.002, <0.001, <0.001, <0.001}
PDW (fL),	12.15 (10.9–13.7)	12.2 (11–13.7)	12.1 (10.8–13.7)	11.9 (10.6–13.4)	<0.001 {0.307, <0.001, 0.039, 0.599, 0.498, <0.001}
RDW-CV,	0.13 (0.12–0.14)	0.13 (0.12–0.14)	0.13 (0.12–0.14)	0.13 (0.13–0.14)	<0.001 {0.287, <0.001, <0.001, 0.033, <0.001, <0.001}

All data are presented as median and interquartile range.

CRP, C Reactive Protein; IL-6, interleukin 6; PCT, procalcitonin; LDH, lactate dehydrogenase; NLR, neutrophil-to-lymphocyte ratio; dNLR, derived neutrophil-to-lymphocyte ratio (ratio of neutrophils to leukocytes − neutrophils); LMR, lymphocyte-to-monocyte ratio; PLR, platelet-to-lymphocyte ratio; NPR, neutrophil-to-platelet ratio; SII, Systemic Immune-Inflammation Index (neutrophils × platelets/lymphocytes); SIRI, systemic inflammation response index (neutrophil count × monocyte count/lymphocyte count); PDW, platelet distribution width; RDW-CV, red blood cell distribution width coefficient of variation.

A significant increase in the inflammatory biomarkers was described during the Delta wave compared to all the other waves, except for the Alpha wave for CRP, PCT, ferritin, D-dimer, LDH, leukocytes, neutrophils, monocytes, basophils, eosinophils, platelets, LMR, NPR, SIRI, platelet distribution width (PDW), and red blood cell distribution width coefficient of variation (RDW-CV).

**Figure 2:**
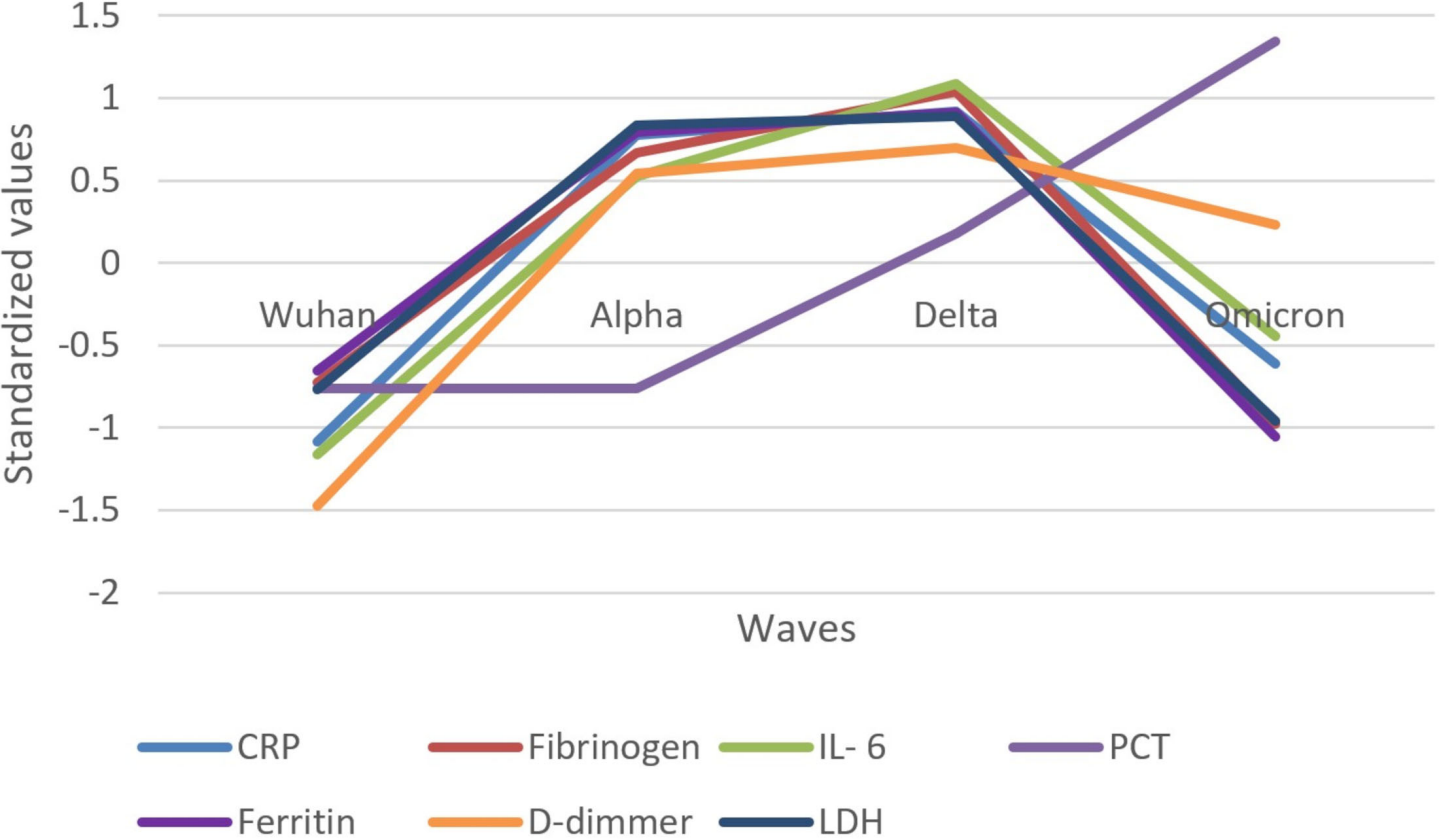
Standardized values of the medians of inflammatory biomarkers evolution by wave. Standardized values were calculated the following way: median marker value per wave – average value of the four median values of the waves/standard deviation of the four median values of the waves; CRP, C Reactive Protein; IL-6, interleukin 6; PCT, procalcitonin; LDH, lactate dehydrogenase.

**Figure 3:**
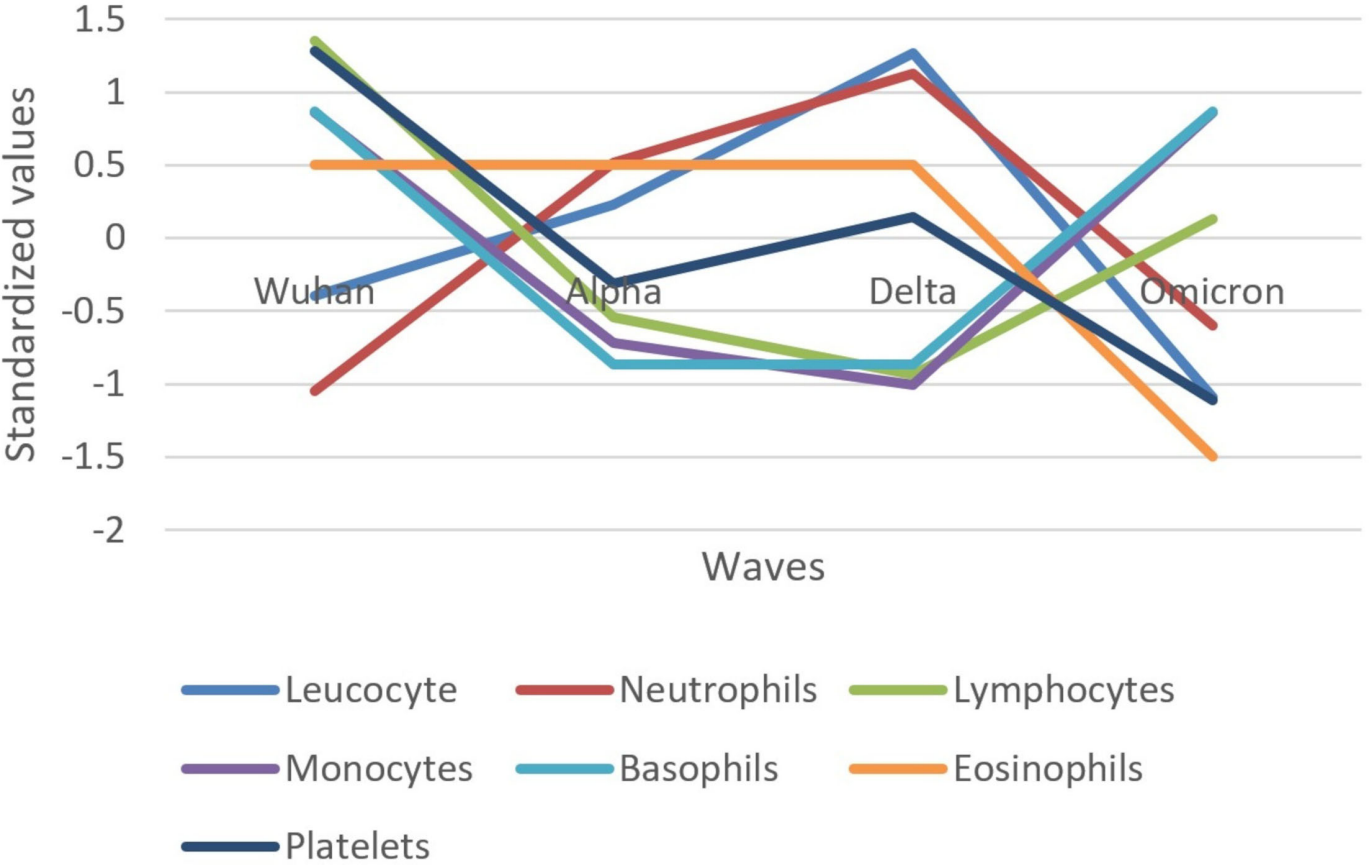
Standardized values of the medians of hematological biomarkers evolution by wave. Standardized values were calculated the following way: median marker value per wave – average value of the four median values of the waves/standard deviation of the four median values of the waves.

**Figure 4:**
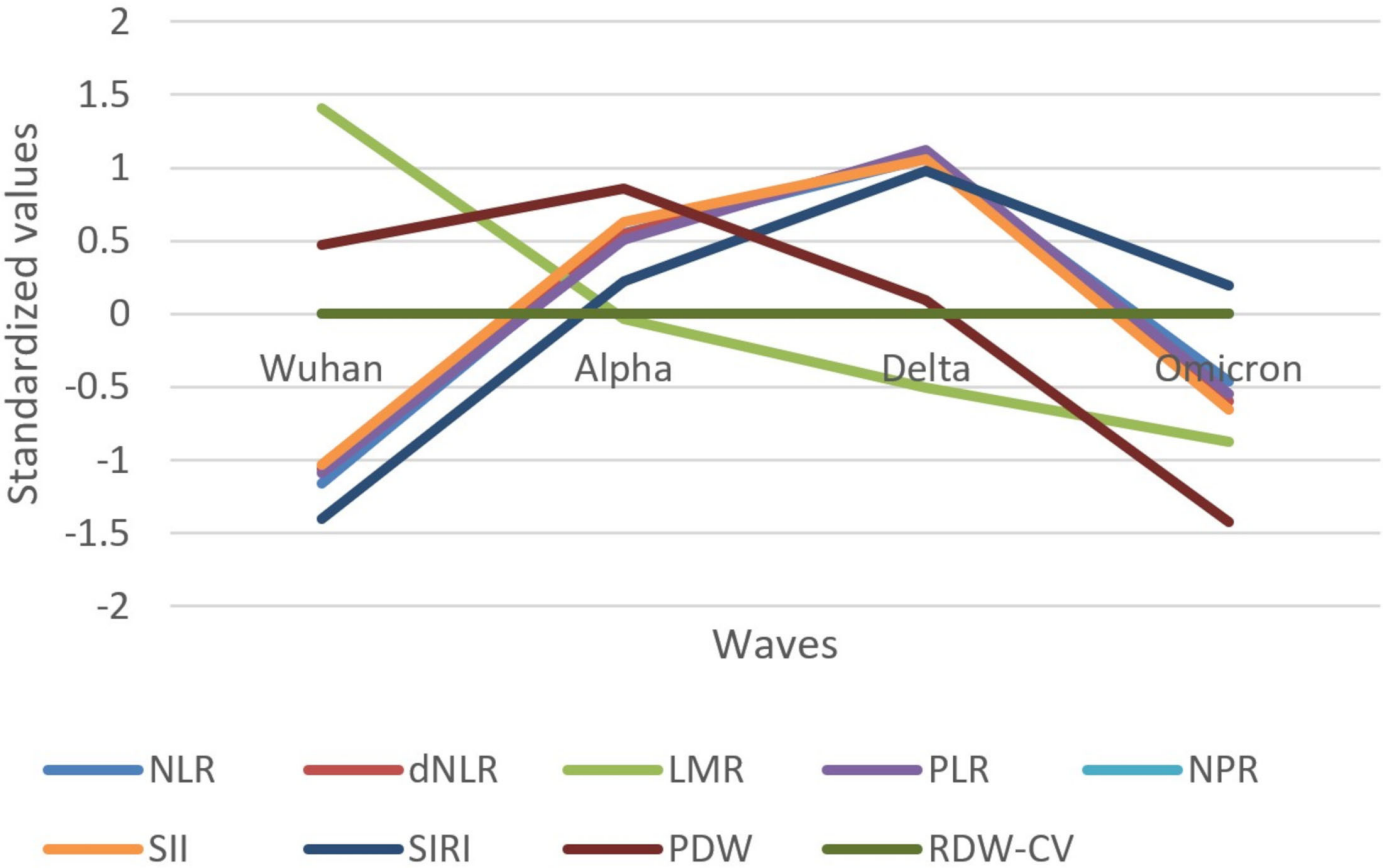
Standardized values of the medians of inflammatory biomarkers evolution by wave. Standardized values were calculated the following way: median marker value per wave – average value of the four median values of the waves/standard deviation of the four median values of the waves; NLR, neutrophile to lymphocyte ratio; dNLR, derived neutrophile to leucocyte ratio (ratio of neutrophils to leukocytes – neutrophils); LMR, lymphocyte to monocyte ratio; PLR, platelets to lymphocyte ratio; NPR, neutrophile to platelets ratio; SII, Systemic Immune-Inflammation Index (neutrophils × platelets/lymphocytes); SIRI, systemic inflammation response index (neutrophil count × monocyte count/lymphocyte count); PDW, platelets distribution width; RDW-CV, Red Blood Cell Distribution Width coefficient of variation.

The evolution of inflammatory biomarkers across different waves was presented graphically, using standardized values, in **Figures 2**–**4**.

Inflammatory biomarkers (CRP, fibrinogen, ferritin, IL-6, D-dimer, and LDH) increased in their median values from the Wuhan to Delta wave and decreased in the Omicron wave, except PCT, which steeply increased from the Alpha to Omicron wave (**Figure 2**).

Concerning hematologic biomarkers, leukocytes and neutrophils increased in their median values from the Wuhan to Delta wave and decreased in the Omicron wave (**Figure 3**). An inverse pattern can be observed for lymphocytes, monocytes, and basophils, which decreased in their median values from the Wuhan to Delta wave and increased in the Omicron wave. Platelets had a different pattern, decreasing from the Wuhan to Alpha wave, increasing in the Delta wave, and finally decreasing in the Omicron wave. Eosinophil values were unchanged in the Wuhan to Delta waves but decreased in the Omicron wave.

Most indexes based on biomarkers (NLR, dNLR, PLR, NPR, SII, and SIRI) showed an increase in standardized values from the Wuhan to Delta wave, followed by a decrease toward the Omicron wave (**Figure 4**). LMR showed a different trend, with values decreasing from the Wuhan wave steadily across subsequent waves. PDW started high in the Wuhan wave, increased in the Alpha wave, and then decreased in the Delta and Omicron waves. RDW-CV had no variation throughout the waves.

The Delta wave generally showed the highest standardized values for most inflammatory biomarkers, indicating a peak in the inflammatory response. The Omicron wave showed a decrease in the inflammatory response compared to the Delta wave, suggesting a reduced severity in terms of inflammation.

Comparing inflammatory biomarkers in severe or critical patients to mild or moderate COVID-19 patients, results were significantly increased for the majority of biomarkers in severe/critical patients (**Table 3**) except lymphocytes, monocytes, and LMR, which were significantly decreased in severe/critical patients. No differences were observed for eosinophils.

**Table 3:** Inflammatory biomarkers in severe or critical patients vs. mild or moderate COVID-19.

Inflammatory biomarkers	Severe/critical (n = 2,709)	Mild/moderate (n = 5,905)	p
CRP (mg/dL)	8.76 (4.25–15.59)	1.27 (0.3–4.37)	<0.001
Fibrinogen (mg/dL)	533.3 (427.94–657.49)	389.43 (311.11–489.21)	<0.001
IL-6 (pg/mL)	31.04 (11.99–73.84)	9.55 (3.59–24.07)	<0.001
PCT (ng/mL)	0.14 (0.05–0.48)	0.05 (0.05–0.18)	<0.001
Ferritin (ng/mL)	499.75 (248.3–961.73)	174.75 (68.53–372.95)	<0.001
D-dimer (mg/L)	0.84 (0.48–1.69)	0.44 (0.27–0.82)	<0.001
LDH (U/L)	347 (252–462.75)	200 (166–257)	<0.001
Leukocytes (10^3^/μL)	7.5 (5.3–10.64)	5.76 (4.4–7.43)	<0.001
Neutrophils (10^3^/μL)	6.04 (3.93–9.01)	3.5 (2.46–5.03)	<0.001
Lymphocytes (10^3^/μL)	0.79 (0.55–1.14)	1.41 (0.97–1.92)	<0.001
Monocytes (10^3^/μL)	0.4 (0.25–0.61)	0.52 (0.38–0.68)	<0.001
Basophils 10^3^/μL	0.01 (0.01–0.02)	0.02 (0.01–0.03)	<0.001
Eosinophils (10^3^/μL)	0.14 (0.09–0.14)	0.14 (0.08–0.14)	0.338
Platelets (10^3^/μL)	221 (164–292)	211 (167–267)	<0.001
NLR	7.63 (4.1–12.89)	2.46 (1.51–4.22)	<0.001
dNLR	4.86 (2.8–7.7)	1.7 (1.11–2.73)	<0.001
LMR	2.06 (1.35–3.19)	2.85 (1.92–3.97)	<0.001
PLR	279.05 (181.79–418.99)	150 (109.32–220)	<0.001
NPR	0.03 (0.02–0.04)	0.02 (0.01–0.02)	<0.001
SII	1,675.63 (823.5–3,248.29)	519.39 (297.14–986.78)	<0.001
SIRI	2.8 (1.36–5.7)	1.23 (0.69–2.3)	<0.001
PDW (fL)	12.2 (10.9–13.8)	12 (10.7–13.5)	<0.001
RDW-CV	0.13 (0.13–0.14)	0.13 (0.12–0.14)	<0.001

All data are presented as median and interquartile range.

CRP, C-reactive protein; IL-6, interleukin 6; PCT, procalcitonin; LDH, lactate dehydrogenase; NLR, neutrophil-to-lymphocyte ratio; dNLR, derived neutrophil-to-lymphocyte ratio (ratio of neutrophils to leukocytes − neutrophils); LMR, lymphocyte-to-monocyte ratio; PLR, platelet-to-lymphocyte ratio; NPR, neutrophil-to-platelet ratio; SII, Systemic Immune-Inflammation Index (neutrophils × platelets/lymphocytes); SIRI, systemic inflammation response index (neutrophil count × monocyte count/lymphocyte count); PDW, platelet distribution width; RDW-CV, red blood cell distribution width coefficient of variation.

Comparing inflammatory biomarkers in patients who died to patients discharged alive, results were significantly increased for the majority of the biomarkers in death outcome (**Table 4**) except lymphocytes, monocytes, basophils, and LMR, which were significantly decreased in severe/critical patients. No differences were observed for eosinophils and platelets.

**Table 4:** Inflammatory markers in function of death outcome.

Inflammatory biomarkers	Death (n = 572)	Survival (n = 8,042)	p
CRP (mg/dL)	11.48 (5.7–20.29)	2.36 (0.5–7.36)	<0.001
Fibrinogen (mg/dL)	525.48 (428.38–680.49)	428.88 (335.2–550.87)	<0.001
IL-6 (pg/mL)	57.38 (25.12–134.56)	13.77 (4.87–35.42)	<0.001
PCT (ng/mL)	0.38 (0.11–1.2)	0.08 (0.05–0.23)	<0.001
Ferritin (ng/mL)	634 (308.62–1,295.72)	229.5 (91.57–509.52)	<0.001
D-dimer (mg/L)	1.25 (0.67–3.16)	0.52 (0.31–1)	<0.001
LDH (U/L)	418 (293–595.5)	222 (175–306)	<0.001
Leukocytes (10^3^/μL)	9.11 (6.09–13.2)	6 (4.53–8.02)	<0.001
Neutrophils (10^3^/μL)	7.91 (5–11.74)	3.93 (2.63–5.89)	<0.001
Lymphocytes (10^3^/μL)	0.64 (0.42–0.93)	1.24 (0.82–1.78)	<0.001
Monocytes (10^3^/μL)	0.4 (0.24–0.59)	0.49 (0.34–0.67)	<0.001
Basophils 10^3^/μL	0.01 (0.01–0.02)	0.02 (0.01–0.03)	<0.001
Eosinophils (10^3^/μL)	0.14 (0.14–0.14)	0.14 (0.08–0.14)	0.082
Platelets (10^3^/μL)	213 (150–282)	214 (168–274)	0.106
NLR	12.44 (6.72–20.25)	3.09 (1.75–6.13)	<0.001
dNLR	7.45 (4.36–10.6)	2.08 (1.26–3.9)	<0.001
LMR	1.69 (1.07–2.78)	2.65 (1.76–3.83)	<0.001
PLR	334.38 (199.26–524.86)	171.26 (117.79–267.97)	<0.001
NPR	0.04 (0.03–0.05)	0.02 (0.01–0.03)	<0.001
SII	2,677.95 (1,192.29–5,147.1)	656.94 (341.62–1,406.5)	<0.001
SIRI	4.75 (2.15–10.01)	1.45 (0.77–2.93)	<0.001
PDW (fL)	12.8 (11.3–14.5)	12 (10.8–13.5)	<0.001
RDW-CV	0.14 (0.13–0.15)	0.13 (0.12–0.14)	<0.001

All data are presented as median and interquartile range.

CRP, C-reactive protein; IL-6, interleukin 6; PCT, procalcitonin; LDH, lactate dehydrogenase; NLR, neutrophil-to-lymphocyte ratio; dNLR, derived neutrophil-to-lymphocyte ratio (ratio of neutrophils to leukocytes − neutrophils); LMR, lymphocyte-to-monocyte ratio; PLR, platelet-to-lymphocyte ratio; NPR, neutrophil-to-platelet ratio; SII, Systemic Immune-Inflammation Index (neutrophils × platelets/lymphocytes); SIRI, systemic inflammation response index (neutrophil count × monocyte count/lymphocyte count); PDW, platelet distribution width; RDW-CV, red blood cell distribution width coefficient of variation.

**Table 5** presents results of the ROC curves concerning inflammatory biomarkers classifying severe or critical vs. mild or moderate COVID-19. The highest AUC was found for CRP, dNLR, LDH, and NLR.

**Table 5:** Receiver operating characteristic classifying severe/critical versus mild/moderate COVID-19, concerning inflammatory biomarkers.

Variable	AUC (95% CI)	Se	Sp	Cut-off
CRP (mg/dL)	0.826 (0.817–0.835)	80.36	69.87	3.41
dNLR	0.822 (0.812–0.832)	72.2	79.27	3.06
LDH (U/L)	0.817 (0.806–0.826)	72.34	76.62	262
NLR	0.812 (0.802–0.822)	72.09	77.26	4.51
SII	0.781 (0.771–0.792)	71.68	72.68	921.42
Lymphocytes (10^3^/μL)	0.758 (0.747–0.77)	72.23	68.73	1.02
Ferritin (ng/mL)	0.752 (0.741–0.764)	75.02	62.34	247.9
PLR	0.749 (0.737–0.761)	66.51	73.07	210.75
Fibrinogen (mg/dL)	0.74 (0.727–0.752)	78.74	57.79	414.34
Neutrophils (10^3^/μL)	0.735 (0.724–0.747)	63.42	73.46	4.90
NPR	0.732 (0.721–0.744)	71.12	64.35	0.02
IL-6 (pg/mL)	0.728 (0.716–0.741)	64.62	68.41	18.35
SIRI	0.713 (0.701–0.725)	63.44	70.78	1.99
D-dimer (mg/L)	0.697 (0.685–0.709)	75.82	53.75	0.47
Basophils 10^3^/μL	0.657 (0.645–0.669)	70.97	57.29	0.01
Leukocytes (10^3^/μL)	0.656 (0.643–0.669)	49.83	76.14	7.5
PCT (ng/mL)	0.638 (0.613–0.662)	65.81	59.6	0.07
LMR	0.633 (0.62–0.646)	69.05	52.29	2.13
RDW-CV	0.631 (0.617–0.645)	44.66	74.65	0.13
Monocytes (10^3^/μL)	0.62 (0.607–0.634)	73.35	48.82	0.39
PDW (fL)	0.532 (0.516–0.548)	42.51	63.19	12.6
Platelets (10^3^/μL)	0.526 (0.512–0.539)	30.06	78.14	276
Eosinophils (10^3^/μL)	0.494 (0.483–0.506)	72.13	32.7	0.14

AUC, area under the curve; Se, sensitivity; Sp, specificity; CRP, C-reactive protein; IL-6, interleukin 6; PCT, procalcitonin; LDH, lactate dehydrogenase; NLR, neutrophil-to-lymphocyte ratio; dNLR, derived neutrophil-to-lymphocyte ratio (ratio of neutrophils to leukocytes − neutrophils); LMR, lymphocyte-to-monocyte ratio; PLR, platelet-to-lymphocyte ratio; NPR, neutrophil-to-platelet ratio; SII, Systemic Immune-Inflammation Index (neutrophils × platelets/lymphocytes); SIRI, systemic inflammation response index (neutrophil count × monocyte count/lymphocyte count); PDW, platelet distribution width; RDW-CV, red blood cell distribution width coefficient of variation.

**Table 6** presents the results of the ROC curves concerning inflammatory biomarkers classifying death outcome vs. survival. The highest area under the curve (AUC) was found for dNLR, NLR, LDH, and NPR.

**Table 6:** Receiver operating characteristic classifying death outcome for COVID-19 patients concerning inflammatory markers.

Variable	AUC (95% CI)	Se	Sp	Cut-off
dNLR	0.842 (0.825–0.858)	84.44	72.42	3.6
NLR	0.836 (0.818–0.853)	86.19	68.12	4.92
LDH (U/L)	0.811 (0.793–0.829)	79.16	68.52	278
NPR	0.802 (0.783–0.82)	75.31	73.85	0.03
CRP (mg/dL)	0.797 (0.779–0.814)	78.85	65.9	5.03
SII	0.791 (0.771–0.812)	74.43	72.46	1,282.59
IL-6 (pg/mL)	0.786 (0.768–0.803)	77.94	64.49	23.22
Lymphocytes (10^3^/μL)	0.774 (0.753–0.794)	67.61	75.17	0.93
Neutrophils (10^3^/μL)	0.769 (0.746–0.791)	66.96	77.36	6.16
SIRI	0.755 (0.73–0.777)	73.91	66.54	2.25
Ferritin (ng/mL)	0.746 (0.726–0.765)	80.04	55.33	270.1
D-dimer (mg/L)	0.743 (0.722–0.763)	63.2	72.65	0.92
PLR	0.733 (0.71–0.755)	62.7	74.65	265.26
PCT (ng/mL)	0.718 (0.692–0.744)	64.48	71.42	0.18
RDW-CV	0.713 (0.694–0.734)	63.04	69.9	0.13
Leukocytes (10^3^/μL)	0.709 (0.685–0.734)	56.12	79.51	8.55
LMR	0.672 (0.646–0.697)	69.93	58.84	1.94
Fibrinogen (mg/dL)	0.655 (0.63–0.678)	71.51	54.09	442.8
Basophils 10^3^/μL	0.624 (0.6–0.647)	0.87	99.86	0.14
Monocytes (10^3^/μL)	0.599 (0.572–0.625)	60.02	57.97	0.43
PDW (fL)	0.587 (0.562–0.613)	47.61	67.52	12.9
Platelets (10^3^/μL)	0.52 (0.493–0.548)	30.47	72.92	267
Eosinophils (10^3^/μL)	0.519 (0.5–0.538)	78.5	31.87	0.14

AUC, area under the curve; Se, sensitivity; Sp, specificity; CRP, C-reactive protein; IL-6, interleukin 6; PCT, procalcitonin; LDH, lactate dehydrogenase; NLR, neutrophil-to-lymphocyte ratio; dNLR, derived neutrophil-to-lymphocyte ratio (ratio of neutrophils to leukocytes − neutrophils); LMR, lymphocyte-to-monocyte ratio; PLR, platelet-to-lymphocyte ratio; NPR, neutrophil-to-platelet ratio; SII, Systemic Immune-Inflammation Index (neutrophils × platelets/lymphocytes); SIRI, systemic inflammation response index (neutrophil count × monocyte count/lymphocyte count); PDW, platelet distribution width; RDW-CV, red blood cell distribution width coefficient of variation.

The results of the multivariate logistic regression models predicting death for specific inflammatory biomarkers and adjusted for age ≥ 65 years, sex, wave (Omicron, Delta, Alpha, and Wuhan), obesity, diabetes, cardiovascular, pulmonary, hepatic, renal, neurologic diseases, cancers, and vaccination status are presented in **Table 7**. The highest OR for death was described for dNLR and NLR, while increased lymphocytes decreased the highest OR death.

**Table 7:** Multivariate logistic regression models predicting death outcome for specific inflammatory markers.

Variable	OR adjusted	(95% CI)	p
dNLR ≥ 3.6	8.46	(6.69–10.82)	<0.001
NLR ≥ 4.92	7.59	(5.94–9.82)	<0.001
LDH ≥ 278 U/L	5.99	(4.8–7.53)	<0.001
NPR ≥ 0.026	5.5	(4.49–6.78)	<0.001
IL-6 ≥ 23.22 (pg/mL)	4.78	(3.88–5.94)	<0.001
SII ≥ 1,282.59	4.61	(3.76–5.66)	<0.001
PCT ≥ 0.18 ng/mL	4.61	(3.64–5.86)	<0.001
CRP ≥ 5.03 mg/dL	4.21	(3.4–5.25)	<0.001
Basophils ≥ 0.137 10^3^/μL	4.15	(1.16–12.88)	0.018
Leukocytes ≥ 8.55 10^3^/μL	3.83	(3.19–4.61)	<0.001
SIRI ≥ 2.25	3.62	(2.97–4.45)	<0.001
Ferritin ≥ 270.1 ng/mL	3.4	(2.71–4.29)	<0.001
D-dimer ≥ 0.92 mg/L	3.12	(2.57–3.8)	<0.001
RDW-CV ≥ 0.13	3.11	(2.47–3.94)	<0.001
PLR ≥ 265.26	3.01	(2.5–3.63)	<0.001
Fibrinogen ≥ 442.8 (mg/dL)	1.92	(1.56–2.38)	<0.001
Neutrophils ≥ 6.155 10^3^/μL	1.78	(1.49–2.15)	<0.001
Eosinophils ≥ 0.14 10^3^/μL	1.71	(1.36–2.18)	<0.001
PDW ≥ 12.9 fL	1.6	(1.32–1.94)	<0.001
Monocytes ≥ 0.43 10^3^/μL	0.62	(0.52–0.75)	<0.001
Platelets ≥ 267 10^3^/μL	0.48	(0.4–0.58)	<0.001
LMR ≥ 1.94	0.45	(0.38–0.55)	<0.001
Lymphocytes ≥ 0.93 10^3^/μL	0.3	(0.24–0.36)	<0.001

OR, odds ratio; CI, confidence interval; CRP, C-reactive protein; IL-6, interleukin 6; PCT, procalcitonin; LDH, lactate dehydrogenase; NLR, neutrophil-to-lymphocyte ratio; dNLR, derived neutrophil-to-lymphocyte ratio (ratio of neutrophils to leukocytes − neutrophils); LMR, lymphocyte-to-monocyte ratio; PLR, platelet-to-lymphocyte ratio; NPR, neutrophil-to-platelet ratio; SII, Systemic Immune-Inflammation Index (neutrophils × platelets/lymphocytes); SIRI, systemic inflammation response index (neutrophil count × monocyte count/lymphocyte count); PDW, platelet distribution width; RDW-CV, red blood cell distribution width coefficient of variation.

**Table 8** presents, for severe or critical patients, the median values of inflammatory biomarkers and indexes in the function of SARS-CoV-2 waves, and the statistical difference in their values between waves. The Omicron wave generally showed a reduction in inflammatory biomarkers compared to earlier waves, indicating potentially less severe inflammation or improved clinical management.

**Table 8:** Inflammatory biomarkers in function of SARS-CoV-2 waves for severe or critical patients.

Wave	Wuhan (n = 650)	Alpha (n = 504)	Delta (n = 857)	Omicron (n = 698)	p {Alpha–Delta, Alpha–Omicron, Delta–Omicron, Alpha–Wuhan, Delta–Wuhan, Omicron–Wuhan}
CRP (mg/dL)	8.93 (4.34–15.66)	9.96 (5.19–16.44)	9.16 (4.55–16.24)	7.32 (3.1–13.99)	<0.001{0.224, <0.001, <0.001, 0.042, 0.356, 0.002}
Fibrinogen (mg/dL)	526.68 (419.08–679.97)	555.45 (442.8–657.49)	560.03 (461–683.66)	467.05 (393.23–591.34)	<0.001{0.173, <0.001, <0.001, 0.198, 0.003, <0.001}
IL-6 (pg/mL)	29.84 (10.99–72.64)	29.33 (11.75–71.13)	28.9 (11.47–62.81)	38.17 (13.52–96.58)	<0.001{0.872, 0.003, <0.001, 1, 1, 0.002}
PCT (ng/mL)	0.12 (0.05–0.44)	0.12 (0.05–0.27)	0.13 (0.05–0.43)	0.2 (0.07–0.82)	<0.001{0.543, <0.001, <0.001, 0.582, 0.984, <0.001}
Ferritin (ng/mL)	596.75 (285.88–1,057.68)	575.5 (314.08–1,040.2)	536.55 (292.65–1,058.48)	305.4 (156.62–630.18)	<0.001{0.837, <0.001, <0.001, 0.945, 0.933, <0.001}
D-dimer (mg/L)	0.88 (0.48–1.9)	0.82 (0.5–1.45)	0.74 (0.44–1.35)	0.96 (0.54–2.17)	<0.001{0.092, 0.002, <0.001, 0.122, <0.001, 0.141}
LDH (U/L)	355 (266–458.5)	394.5 (311.75–511.75)	371 (281–497)	253 (200–353)	<0.001{0.002, <0.001, <0.001, <0.001, 0.014, <0.001}
Leukocytes (10^3^/μL)	8.24 (5.85–11.33)	7.45 (5.24–10.62)	7.04 (5.02–9.9)	7.5 (5.25–10.8)	<0.001{0.119, 0.965, 0.047, 0.019, <0.001, 0.014}
Neutrophils (10^3^/μL)	6.73 (4.36–9.65)	6.15 (3.96–9.02)	5.69 (3.88–8.57)	5.77 (3.74–9.17)	<0.001{0.246, 0.419, 0.821, 0.104, <0.001, 0.003}
Lymphocytes (10^3^/μL)	0.82 (0.55–1.2)	0.79 (0.56–1.1)	0.75 (0.54–1.06)	0.82 (0.55–1.27)	0.002{0.289, 0.186, 0.002, 0.37, 0.017, 0.628}
Monocytes (10^3^/μL)	0.42 (0.29–0.63)	0.38 (0.23–0.58)	0.36 (0.23–0.54)	0.44 (0.28–0.67)	<0.001{0.157, <0.001, <0.001, 0.006, <0.001, 0.564}
Basophils 10^3^/μL	0.01 (0.01–0.02)	0.01 (0.01–0.02)	0.01 (0.01–0.02)	0.01 (0.01–0.02)	<0.001 {0.89, 0.002, 0.002, 0.01, 0.013, 0.695}
Eosinophils (10^3^/μL)	0.14 (0.14–0.14)	0.14 (0.14–0.14)	0.14 (0.14–0.14)	0.14 (0.01–0.14)	<0.001{0.519, <0.001, <0.001, 0.361, 0.053, <0.001}
Platelets (10^3^/μL)	238 (174–313)	230 (176–307)	225 (165–294)	201 (147–257)	<0.001{0.224, <0.001, <0.001, 0.608, 0.037, <0.001}
NLR	7.93 (4.16–14.12)	8.04 (4.53–12.64)	7.85 (4.51–12.33)	6.63 (3.57–12.39)	0.005{1, 0.022, 0.019, 1, 1, 0.011}
dNLR	5.12 (2.73–8.08)	5.02 (2.99–7.62)	5.03 (3.16–7.55)	4.14 (2.28–7.52)	<0.001{1, <0.001, <0.001, 1, 1, 0.001}
LMR	1.97 (1.29–3.12)	2.12 (1.42–3.23)	2.14 (1.43–3.27)	2 (1.24–3.15)	0.052{1, 0.184, 0.115, 0.229, 0.216, 1}
PLR	280.21 (180.39–441.03)	295.24 (195.05–432.03)	297.52 (200–426.32)	237.06 (152.19–368.87)	<0.001{0.998, <0.001, <0.001, 0.44, 0.277, <0.001}
NPR	0.03 (0.02–0.04)	0.03 (0.02–0.04)	0.03 (0.02–0.04)	0.03 (0.02–0.04)	<0.001{1, 0.006, 0.002, 0.162, 0.121, 0.186}
SII	1,878.97 (862.88–3,726.85)	1,850.44 (943.28–3,214.2)	1,716.58 (859.54–3,231.43)	1,319.2 (630.42–2,694.62)	<0.001{0.654, <0.001, <0.001, 0.778, 0.316, <0.001}
SIRI	3.26 (1.52–6.52)	2.86 (1.35–5.59)	2.47 (1.34–5.09)	2.78 (1.29–6.01)	0.007{0.291, 1, 0.223, 0.16, 0.004, 0.22}
PDW (fL)	12.35 (11–13.8)	12.2 (11.1–13.7)	12.1 (10.8–13.7)	12.3 (10.6–14.1)	0.33 {0.62, 0.95, 0.889, 0.831, 0.61, 0.904}
RDW-CV	0.13 (0.13–0.14)	0.13 (0.13–0.14)	0.13 (0.12–0.14)	0.14 (0.13–0.15)	<0.001{0.553, <0.001, <0.001, 0.015, <0.001, <0.001}

All data are presented as median and interquartile range.

CRP, C-reactive protein; IL-6, interleukin 6; PCT, procalcitonin; LDH, lactate dehydrogenase; NLR, neutrophil-to-lymphocyte ratio; dNLR, derived neutrophil-to-lymphocyte ratio (ratio of neutrophils to leukocytes − neutrophils); LMR, lymphocyte-to-monocyte ratio; PLR, platelet-to-lymphocyte ratio; NPR, neutrophil-to-platelet ratio; SII, Systemic Immune-Inflammation Index (neutrophils × platelets/lymphocytes); SIRI, systemic inflammation response index (neutrophil count × monocyte count/lymphocyte count); PDW, platelet distribution width; RDW-CV, red blood cell distribution width coefficient of variation.

**Figure 5:**
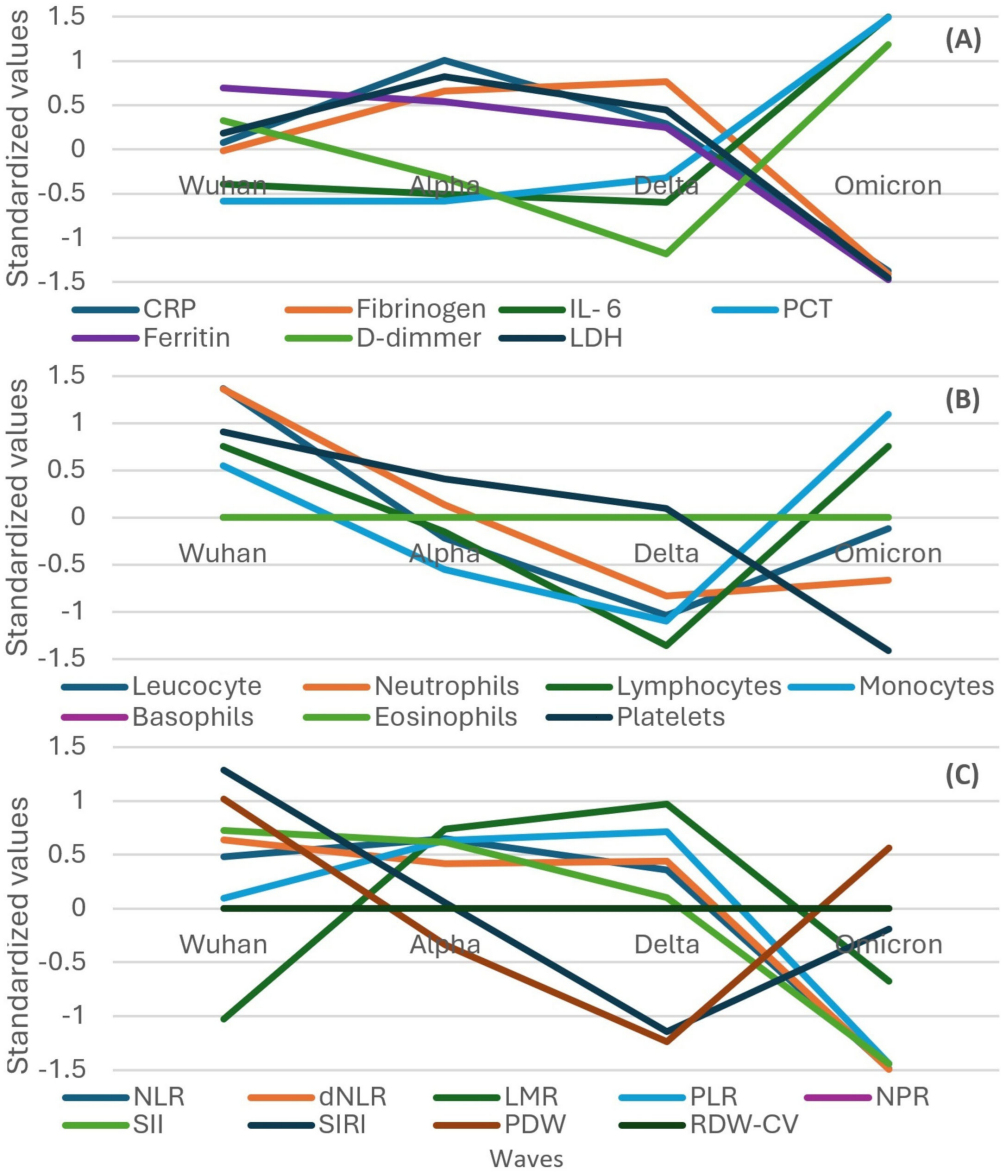
Standardized values of the medians of inflammatory biomarkers evolution by wave in the subgroup of severe or critical participants. Standardized values were calculated the following way: median marker value per wave – average value of the four median values of the waves/standard deviation of the four median values of the waves. **(A)** presents: CRP, fibrinogen, IL-6, PCT, Ferritin, D-Dimmer, LDH; **(B)** presents: leucocyte, neutrophils, lymphocytes, monocytes, basophils, eosinophils, platelets counts; **(C)** presents: NLR, dNLR, LMR, PLR, NPR, SII, SIRI, PDW, RDW-CW. CRP, C Reactive Protein; PCT, procalcitonin; LDH, lactate dehydrogenase; NLR, neutrophile to lymphocyte ratio; dNLR, , derived neutrophile to leucocyte ratio (ratio of neutrophils to leukocytes – neutrophils); LMR, lymphocyte to monocyte ratio; PLR, platelets to lymphocyte ratio; NPR, neutrophile to platelets ratio; SII, Systemic Immune-Inflammation Index (neutrophils × platelets/lymphocytes); SIRI, systemic inflammation response index (neutrophil count × monocyte count/lymphocyte count); PDW, platelets distribution width; RDW-CV, Red Blood Cell Distribution Width coefficient of variation.

Some biomarkers like IL-6 and D-dimer had a different trend in the subgroup of severe or critical patients than in all the participants, which decreased from the Wuhan to Delta wave and increased in the Omicron wave (**Figure 5A** compared to **Figure 2**).

In contrast with the evolution of all the participants, leukocytes and neutrophils had a descending trend from the Wuhan to Delta wave in the subgroup of severe or critical patients (**Figure 5B** compared to **Figure 3**).

A different trend compared to that in all the participants was observed in the subgroup of severe or critical participants for PDW and SIRI, which showed a decreasing trend from the Wuhan to Delta wave and then an increasing one for the Omicron wave. Also, the LMR increased from the Wuhan to Delta wave and then decreased in the Omicron wave (**Figure 5C** compared to **Figure 4**).

Inflammatory biomarkers in the function of SARS-CoV-2 waves, for COVID-19 patients who died, are presented in **Table 9**, and no significant difference between waves was described for most of the investigated biomarkers.

**Table 9:** Inflammatory biomarkers in function of SARS-CoV-2 waves for COVID-19 patients who died.

Inflammatory biomarkers	Wuhan (n = 153)	Alpha (n = 105)	Delta (n = 178)	Omicron (n = 136)	p {Alpha–Delta, Alpha–Omicron, Delta–Omicron, Alpha–Wuhan, Delta–Wuhan, Omicron–Wuhan}
CRP (mg/dL),	10.57 (5.14–19.41)	11.45 (7.64–22.66)	13.46 (6.18–21.53)	11.46 (5.23–18.09)	0.149 {1, 0.318, 0.359, 0.438, 0.315, 0.884}
Fibrinogen (mg/dL)	525.64 (417.21–679.97)	533.53 (451.74–663.91)	546.43 (462.55–711.88)	474.76 (384.7–605.01)	0.003 {0.513, 0.081, 0.002, 0.505, 0.13, 0.234}
IL-6 (pg/mL)	59.72 (24.52–131.04)	54.25 (25.69–121.51)	52.63 (26.53–101.97)	73.36 (27.42–296.21)	0.115 {1, 0.27, 0.167, 1, 1, 0.198}
PCT (ng/mL)	0.44 (0.11–1.55)	0.22 (0.1–0.82)	0.33 (0.1–1.05)	0.4 (0.15–1.32)	0.26 {0.953, 0.743, 0.447, 0.521, 0.496, 0.932}
Ferritin (ng/mL)	678.7 (332–1,319.1)	809.4 (357.6–1,400.3)	660.75 (314.5–1,283.55)	469 (262.4–1,200.5)	0.214 {0.891, 0.381, 0.346, 0.887, 1, 0.387}
D-dimer (mg/L)	1.32 (0.72–3.23)	1.23 (0.73–2.3)	0.97 (0.51–1.8)	2.1 (0.78–5.12)	<0.001 {0.054, 0.063, <0.001, 0.782, 0.013, 0.132}
LDH (U/L)	415 (286–564)	430 (356–612)	467 (304.25–686.25)	357 (243.5–510)	<0.001 {0.92, <0.001, <0.001, 0.207, 0.209, 0.045}
Leukocytes (10^3^/μL)	10.45 (7.11–13.59)	8.7 (5.95–12.7)	8.29 (5.5–12.18)	9.16 (6.24–13.68)	0.014 {0.713, 0.485, 0.216, 0.115, 0.015, 0.393}
Neutrophils (10^3^/μL)	9.09 (5.83–12.46)	7.58 (4.82–10.93)	7.23 (4.54–10.68)	7.98 (5.08–11.66)	0.027 {0.757, 0.825, 0.434, 0.192, 0.023, 0.256}
Lymphocytes (10^3^/μL)	0.63 (0.41–0.92)	0.65 (0.44–0.96)	0.64 (0.43–0.92)	0.66 (0.38–0.97)	0.995 {1, 1, 1, 1, 1, 1}
Monocytes (10^3^/μL)	0.4 (0.28–0.6)	0.38 (0.2–0.55)	0.39 (0.22–0.55)	0.42 (0.25–0.66)	0.137 {1, 0.481, 0.339, 0.248, 0.268, 0.969}
Basophils 10^3^/μL	0.01 (0.01–0.02)	0.01 (0.01–0.02)	0.01 (0.01–0.02)	0.01 (0.01–0.02)	0.224 {1, 1, 1, 0.572, 0.499, 0.267}
Eosinophils (10^3^/μL)	0.14 (0.14–0.14)	0.14 (0.14–0.14)	0.14 (0.14–0.14)	0.14 (0.01–0.14)	<0.001 {0.825, <0.001, <0.001, 0.867, 0.515, <0.001}
Platelets (10^3^/μL)	228.5 (168.75–305.75)	192 (135–268)	214 (155.5–277.75)	193 (131.5–263)	0.017 {0.393, 0.878, 0.262, 0.067, 0.258, 0.024}
NLR	14.22 (7.41–24.92)	12.24 (7.51–17.2)	11.5 (6.57–18.18)	11.49 (6.6–22.24)	0.191 {1, 1, 1, 0.355, 0.324, 0.441}
dNLR	8.37 (5.17–11.56)	7.19 (4.97–9.12)	7.09 (4.19–9.65)	7.53 (4.11–11.53)	0.109 {0.955, 1, 1, 0.266, 0.137, 0.346}
LMR	1.63 (1.02–2.77)	1.9 (1.26–2.79)	1.67 (1.12–2.93)	1.67 (0.88–2.6)	0.239 {0.672, 0.366, 0.534, 0.447, 0.846, 0.76}
PLR	374.64 (204.51–559.44)	313.43 (189.08–485.11)	333.03 (215.39–499.55)	303.22 (190.13–530.03)	0.33 {0.656, 1, 0.918, 0.528, 0.746, 0.751}
NPR	0.04 (0.03–0.06)	0.03 (0.03–0.06)	0.03 (0.02–0.05)	0.04 (0.03–0.06)	0.117 {0.606, 0.725, 0.182, 0.778, 0.259, 0.779}
SII	3,364.08 (1,521.18–5,872.84)	2,567.82 (1,112.64–4,231.45)	2,560.29 (1,067.23–5,002.64)	2,392.03 (1,120.65–5,018.26)	0.05 {1, 1, 1, 0.069, 0.091, 0.157}
SIRI	5.28 (2.29–10.94)	4.48 (2.02–7.59)	4.14 (1.95–9.54)	5.17 (2.38–10.34)	0.154 {1, 0.472, 0.359, 0.265, 0.452, 0.907}
PDW (fL)	12.85 (11.43–14.28)	12.7 (11.6–14.97)	12.5 (11.3–14.4)	12.9 (10.6–14.33)	0.412 {1, 0.611, 1, 0.796, 0.871, 0.685}
RDW-CV	0.14 (0.13–0.15)	0.14 (0.13–0.15)	0.14 (0.13–0.15)	0.14 (0.14–0.16)	<0.001 {1, 0.003, <0.001, 0.083, 0.033, 0.163}

All data are presented as median and interquartile range.

CRP, C-reactive protein; IL-6, interleukin 6; PCT, procalcitonin; LDH, lactate dehydrogenase; NLR, neutrophil-to-lymphocyte ratio; dNLR, derived neutrophil-to-lymphocyte ratio (ratio of neutrophils to leukocytes − neutrophils); LMR, lymphocyte-to-monocyte ratio; PLR, platelet-to-lymphocyte ratio; NPR, neutrophil-to-platelet ratio; SII, Systemic Immune-Inflammation Index (neutrophils × platelets/lymphocytes); SIRI, systemic inflammation response index (neutrophil count × monocyte count/lymphocyte count); PDW, platelet distribution width; RDW-CV, red blood cell distribution width coefficient of variation.

**Figure 6:**
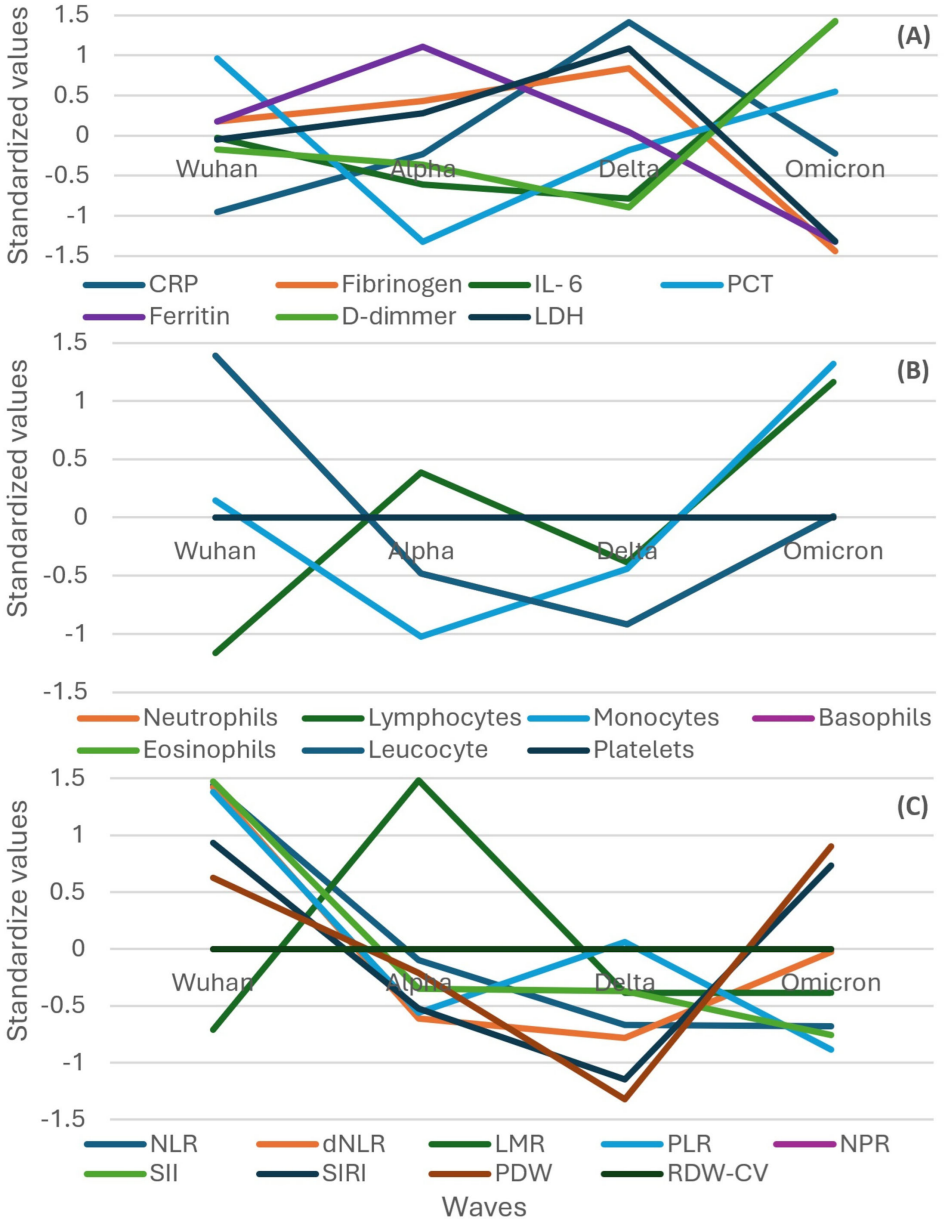
Standardized values of the medians of inflammatory biomarkers evolution by wave in the subgroup of participants that evolved to death. Standardized values were calculated the following way: median marker value per wave – average value of the four median values of the waves/standard deviation of the four median values of the waves. **(A)** presents: CRP, fibrinogen, IL-6, PCT, Ferritin, D-Dimmer, LDH; **(B)** presents: neutrophils, lymphocytes, monocytes, basophils, eosinophils, leucocyte, platelets counts; **(C)** presents: NLR, dNLR, LMR, PLR, NPR, SII, SIRI, PDW, RDW-CW. CRP, C Reactive Protein; PCT, procalcitonin; LDH, lactate dehydrogenase; NLR, neutrophile to lymphocyte ratio; dNLR, derived neutrophile to leucocyte ratio (ratio of neutrophils to leukocytes – neutrophils); LMR, lymphocyte to monocyte ratio; PLR, platelets to lymphocyte ratio; NPR, neutrophile to platelets ratio; SII, Systemic Immune-Inflammation Index (neutrophils × platelets/lymphocytes); SIRI, systemic inflammation response index (neutrophil count × monocyte count/lymphocyte count); PDW, platelets distribution width; RDW-CV, Red Blood Cell Distribution Width coefficient of variation.

In the subgroup of patients who died, the evolution of IL-6 and D-dimers had a different trend than that in all the participants, which decreased from the Wuhan to Delta wave and increased in the Omicron wave, an evolution similar to that of severe or critical participants (**Figure 6A** compared to **Figures 2**, **5A**).

In contrast with the evolution of all the participants, leukocytes and neutrophils had a descending trend from the Wuhan to Delta wave in the subgroup of patients who died, in a similar pattern observed in severe or critical patients (**Figure 6B** compared to **Figures 3**, **5B**).

In contrast with the evolution of all the participants, an almost inversed pattern was observed for SIRI, dNLR, PDW, NLR, and SII, which had a descending trend from the Wuhan to Delta wave in the subgroup of patients who died, in a similar but more pronounced way with the pattern observed in severe or critical patients (**Figure 6C** compared to **Figures 4**, **5C**).

## 4 Discussion

Secondary to SARS-CoV-2 infection, an uncontrolled innate immune response from the host can cause the hyperinflammatory state, which can affect different body systems and lead to increased levels of proinflammatory cytokines like IL-1, IL-6, TNF-α, and other acute-phase markers like ferritin, D-dimer, or CRP (23). This “cytokine storm” plays a pivotal role in the development of severe COVID-19, including pulmonary damage, microvascular thrombosis, renal complications, or shock (24).

In the present observational study of a large cohort of 8,614 COVID‐19 hospitalized patients, we evaluated the relationship between inflammatory biomarkers and outcomes in hospitalized COVID-19 patients across four pandemic waves.

The results of the study highlight significant variations in patients’ demographics, comorbidities, disease severity, and outcomes across different pandemic waves. A notable trend is the increasing age of hospitalized patients with each successive wave, especially in the Omicron wave, alongside a predominance of female hospitalizations, except during the Alpha wave.

Comorbidities rose from the Wuhan to Delta wave but decreased during the Omicron wave, paralleling trends observed in disease severity and mortality, except neurologic and renal diseases and cancer, which increased during the Omicron wave, which may be related to increased age.

As reported by the WHO (14) and in line with international data, the Delta wave emerged in our study population as particularly severe, characterized by the longest hospitalizations and the highest percentage of severe/critical cases and deaths.

The inflammatory biomarkers during the pandemic wave showed different trends, but all were less increased during the Omicron wave except PCT, which continuously increased through to the Omicron wave. The Delta wave was characterized by a significant elevation in inflammatory biomarkers compared to the other waves.

Hematologic biomarkers also reflected these trends, with leukocytes and neutrophils peaking during the Delta wave. The Omicron wave, however, marked a reduction in these inflammatory responses, suggesting a shift toward a less severe clinical profile. In the severe/critical group and the patients who died group, D-dimers, IL-6, and PCT increased also during the Omicron wave. A study on 300 hospitalized COVID-19 patients comparing inflammatory biomarkers between three waves (Alpha, Delta, and Omicron) showed that the Omicron variant presented higher D-dimer levels (p = 0.04), but discordant to our results, no other significant differences were found in inflammatory biomarkers among the three variants (15). Park et al., in a study on 29,075 veterans, compared inflammatory biomarkers between three waves and showed that veterans infected with Omicron showed milder inflammatory responses and lower mortality than with the Alpha and Delta variants (17). Vasbinder et al., in an international study on 3,099 COVID-19 patients comparing the Omicron to pre-Omicron, showed that soluble urokinase plasminogen activator receptor (SuPAR) was the most important predictor of death and mechanical intubation outcome with the highest AUC (0.712), followed by CRP (AUC = 0.642), ferritin (AUC = 0.619), IL‐6 (AUC = 0.614), D‐dimer (AUC = 0.606), and lastly procalcitonin (AUC = 0.596); no other biomarker was investigated (18).

### 4.1 C-reactive protein

When evaluating the inflammatory biomarkers for predicting the disease severity, the serum CRP levels at hospital admission best discriminated between severe/critical and non-severe cases (cut-off 3.41 mg/dL, AUC = 0.826), followed by dNLR, LDH, and NLR. Paranga et al. identified CRP, among all investigated analytes, to best discriminate between severe and non-severe forms of the disease (cut-off = 7.607 mg/dL, AUC = 0.72) (16). An increase in median values of CRP was described from the Wuhan to Alpha and Delta waves with a decrease in the Omicron wave in the whole study group but also in the severe/critical subgroup or in patients who died. Interestingly, a study published on seven pandemic waves in Madrid, Spain, on 5,510 patients hospitalized between 4 March 2020 and 31 December 2022 found the highest median values during the first wave (10.9 mg/dL) (25). The high differences in the median values with our Wuhan group (1.31 mg/dL) may be explained by inclusion in our study of all hospitalized patients, irrespective of severity form. During the Wuhan wave, a high number of asymptomatic patients were hospitalized (11.79% of the subgroup of the Wuhan wave), as imposed by Romanian legislation at that time. The values described for CRP in the Spanish study during the first wave are comparable with the median values described in our subgroup of severe/critical patients during the Wuhan wave (8.93 mg/dL) or in the subgroup of patients who died during the Wuhan wave (10.57 mg/dL).

### 4.2 Ferritin

Ferritin plays a role in the pathogenesis of inflammatory diseases with high levels found in anti-phospholipid syndrome, macrophage activation syndrome, adult-onset Still’s disease, and septic shock, as well as in viral diseases like dengue fever, influenza H5N1, and COVID-19 (26). An Italian multicentric study on 200 COVID-19 hospitalized patients found on-admission mean ferritin levels of 1,650.93 ± 2,396.39 ng/mL (27). In our study, the highest median ferritin level at admission associated with the Delta wave was 399.55 ng/mL, the cut-off for severe/critical COVID was 247.9 ng/mL, and the median ferritin level in severe/critical COVID-19 patients was 499.75 ng/dL. A meta-analysis of 189 observational studies with data from 57,563 COVID-19 patients published in the first year of the pandemic reported a significant difference in mean ferritin levels of 606.37 ng/mL between survivors and non-survivors (28), while in our study, the difference between median values in the two subgroups was 404.5 ng/mL. A study including 141 patients with COVID-19 reported serum ferritin >500 μg/L in all severe patients on admission, and ROC curve analysis confirmed the excellent prognostic accuracies of serum ferritin (cut-off 500 μg/L) to discriminate patients with severe clinical conditions (AUC = 0.939; p < 0.001) (29). Our study found a cut-off in the ROC curve for severe/critical COVID of 247.9 ng/mL (AUC = 0.752) and a cut-off in the ROC curve for progression to death of 270.1 ng/mL (AUC = 0.746). The evolution during waves was similar to the CRP pattern in the whole study group (increase from the Wuhan to Alpha wave and then the Delta wave and a decrease in the Omicron wave), while the subgroup of severe/critical patients had a descending trend from the Wuhan to Alpha wave and then the Delta (but not significantly statistic) and Omicron waves (p < 0.001), and the subgroup of patients who died had an increasing trend from the Wuhan to Alpha wave and then a decreasing trend to the Omicron wave, but the difference was not statistically significant. Irrespective of the subgroup, the lowest median values were found in the Omicron wave, similar to the study published by San Martín-López et al., who found median values between 412 and 610 ng/mL during the first six waves with a decrease in the median value of 196 ng/mL during the seventh wave (25).

### 4.3 Interleukin 6

Increased IL-6 level is regarded as a marker of systemic inflammation and unfavorable prognosis in COVID-19 (3). IL-6, a cytokine with both anti-inflammatory and proinflammatory properties, plays an important role in developing SARS-CoV-2, as a proinflammatory cytokine, but also influences the initiation of coagulation (30) and activate the hepatocytes to induce CRP and fibrinogen secretion (2). Studies published at the beginning of the pandemic supported a cut-off value greater than 55 pg/mL for serum IL-6 when identifying patients at high risk for severe COVID-19 (31), and mortality was found to be associated with an IL-6 value of ≥100 pg/mL (32). The values are higher than in our study, which showed a median IL-6 of 31.04 pg/dL in severe/critical patients and a median of 57.38 (pg/mL) in patients who died. ROC analyses classifying severe/critical versus mild/moderate COVID showed an IL-6 cut-off of 18.35 pg/mL with AUC = 0.728, with a slightly higher cut-off value for death of 23.22 pg/mL and an AUC of 0.786. Quite close to our results, Paranga et al. found an IL-6 cut-off for death of 14.86 pg/mL and AUC = 0.752 (16), Regarding the whole study group, we described an increase in IL-6 values from the Wuhan to Alpha and Delta waves, with a decrease in the Omicron wave, quite close to the pattern from the study published on seven pandemic waves in Madrid, Spain (25). An Italian study analyzed the behavior of IL-6 on 181 patients and showed that the magnitude of IL-6 increases was notably lower in the second and third waves compared to the initial wave (33). We found a similar pattern in the median of IL-6 in the subgroup of severe/critical patients for the first three waves but an increase in IL-6 during the Omicron wave. For patients who died, no statistically significant differences between waves were found regarding IL-6.

### 4.4 Lactate dehydrogenase

LDH is an intracellular enzyme that plays a role in energy production, with the highest concentration found in the heart, lungs, liver, and skeletal muscle (34), and an increase in LDH activity in severe COVID-19 may be related to cell damage as well as impaired blood flow and oxygen delivery (35). Elevated serum LDH levels have been widely reported in COVID-19 cases and were predominantly higher in severe patients. According to a meta-analysis published in 2020 on 3,117 hospitalized COVID-19 patients, the mean value of LDH in severe patients was 1.54 times higher than in non-severe cases (344.48 U/L vs. 224.20 U/L) (36). The median values identified in patients from our cohort were 347 U/L in severe patients vs. 200 U/L in non-severe patients. The median value in patients with severe/critical COVID-19 was 371 U/L in the Delta wave and 253 U/L in the Omicron wave, quite close to the results published by Paranga et al. (362 U/L in Delta vs. 331.5 U/L in the Omicron wave) (16). They also found a cut-off in the ROC curve for the evolution toward death of 379.50 U/L and AUC = 0.809 (the best predictor of mortality), while in our study, in both ROC curves classifying severe/critical vs. mild/moderate COVID-19 or death outcome vs. survival, LDH was found with AUC above 0.8 in both analyses and with a cut-off of 278 U/L for death. Regarding the evolution through the pandemic, LDH values increased from the Wuhan to Alpha and Delta waves, with a decrease in the Omicron wave (statistically significant). Martinot et al., comparing 2,932 hospitalized patients pre-Omicron and during the Omicron wave, showed a decrease in median LDH values in the Omicron wave (283 U/L vs. 233 U/L) (37).

### 4.5 Procalcitonin

The production and release into the circulation of procalcitonin from extrathyroidal tissues are amplified during bacterial infections, actively sustained by enhanced concentrations of IL-1β, TNF-α, and IL-6, while the synthesis of this biomarker is inhibited by IFN-γ, whose concentration increases during viral infections (38). SARS-CoV-2 can trigger an inflammatory cascade via the release of proinflammatory cytokines, such as IL-1β and IL-6, after activating Toll-like receptors, which are also known to stimulate the release of PCT (39). A study performed before the pandemic on severe respiratory viral infections showed that PCT rises during pure viral infections with disease severity in the absence of bacterial pneumonia (39). Slightly increased PCT levels (normal range less than 0.1 ng/mL) were observed in severe COVID-19 patients (mean 0.1 ng/mL) compared to non-severe patients (mean 0.05 ng/mL) (40), quite similar to our results (median 0.14 ng/mL for severe/critical vs. 0.05 ng/mL for mild/moderate). Increased PCT values were associated with a nearly fivefold higher risk of severe SARS-CoV-2 infection in a study published in March 2020, even if bacterial co-infection could not be ruled out (41). Our results showed median values much lower than in bacterial infections and sepsis, with the highest values in patients who died during the Wuhan wave (median 0.44 ng/mL). Regarding the pattern of evolution during the pandemic waves, PCT showed an interesting pattern of increased median values from the Alpha to Omicron wave in the whole study group as well as in the subgroup of severe/critical patients. A more delayed hospitalization during the Omicron wave, bacterial co-infection, and older age of the patients with impaired renal excretion of PCT could explain for increased values during the Omicron wave.

### 4.6 D-dimer

Elevated D-dimer is common in COVID-19 patients and reflects the higher thromboembolic risk in severe cases. A meta-analysis including 5,872 COVID-19 patients published in the first pandemic year showed that higher D-dimer concentrations were associated with severity and death (42). Regarding the evolution during the pandemic waves, an increase from the Wuhan to Alpha and Delta waves was described in our study group with a decrease in the Omicron wave, while the subgroup of severe/critical patients and patients who died had the highest mean values during the Omicron wave. A similar pattern was described in the study of Homen-Fernandez et al. (15), with an increase from the Alpha to Delta and Omicron waves, significantly statistically (from 0.840 mg/L to 0.970 and 1.099 mg/L).

A case–control study on 248 COVID-19 patients showed that D-dimer > 2 mg/L at admission was an independent risk factor for increased mortality (OR 10.7, 95% CI: 1.10−94.38) (43). In our multivariate model predicting death, D-dimer > 0.92 mg/L had an adjusted OR (aOR) of 3.12 with an AUC of 0.743.

### 4.7 Systemic inflammatory indexes

NLR reflects the online dynamic relationship between innate (neutrophils) and adaptive cellular immune response (lymphocytes) during illness. NLR, influenced by conditions like age, chronic diseases like coronary heart disease, stroke, diabetes, obesity, psychiatric diagnosis, cancer, anemia, and stress, is also a very sensitive indicator of infection, inflammation, and sepsis (44, 45). Evidence indicates that the dysregulated myeloid response to SARS-CoV-2 extends to neutrophils in severe COVID-19, while the occurrence of profound lymphopenia in patients with severe COVID-19 is well-established (23). The neutrophil/lymphocyte ratio has been reported to be prognostic of respiratory failure and death in COVID-19. Two recent large meta-analyses showed that severe and non-survivor COVID-19 patients had higher on-admission NLR levels compared to non-severe and survivor patients (46, 47). NLR cut-off ≥4.5 with an AUC for disease severity of 0.85 was found by Li et al. (48), quite similar to our results (the cut-off for severe/critical disease was calculated at 4.5 with AUC = 0.812). Paranga et al. found a cut-off of 8.21 for severe/critical with an AUC = 0.692 (16). A Romanian study on 108 COVID-19 hospitalized patients showed that the optimal cut-off for mortality for NLR, dNLR, LMR, and SIRI was 9.1, 9.6, 0.69, and 2.2, respectively (48).

dNLR with a cut-off of 3.6 was found in our ROC analysis to assess death outcome with the highest AUC (0.842), while Ghobadi et al. in a study on 1,792 COVID-19 patients found a cut-off of 5.83 and an AUC of 0.85 (49). dNLR presented also in our study the second highest AUC for severe/critical disease at a cut-off of 3.05, and in the multivariate logistic regression model, it was the best death predictor (aOR = 8.46).

The cut-off for death for SIRI was 2.25 (AUC = 0.755) and for NPR was 0.026 (AUC = 0.802) similar to a smaller Romanian study performed on 108 patients hospitalized with COVID-19 in 2021 (48), and that found for NLR, dNLR, and SIRI was a cut-off for death of 9.1 (AUC = 0.788), 9.6 (AUC = 0.812), and 2.2 (AUC = 0.763), respectively.

### 4.8 Limitations and strengths

This retrospective study has several limitations that should be acknowledged. First, the data were collected from a single healthcare unit, which may limit the generalization of findings to other populations and healthcare settings. Variations in healthcare practices, patient demographics, and local COVID-19 waves could affect the applicability of these results elsewhere. Additionally, the study period spanned multiple waves of the COVID-19 pandemic, each characterized by different viral variants, public health measures, and treatment protocols. These variations make it challenging to isolate the effects of the virus itself from external factors such as changes in clinical management or the availability of vaccines. The full potential impact of vaccination status on the study outcomes was not fully controlled, as the timing and type of vaccination were not included in the multivariate analysis. Moreover, we separated the study interval into waves based on national data on the predominance of different VOCs, but as no individual VOC identification was available and the co-circulation of different VOCs existed, this limited the absolute association of patients to the different VOCs. Changes in treatment protocols could not have influenced the results of inflammatory biomarkers, as we included in the analysis the inflammatory biomarkers performed at admittance and COVID-19 treatment was started after laboratory samples were collected. However, increased clinical experience and changes in treatment protocols with the introduction of more effective treatments, such as monoclonal antibodies, antivirals, and immunomodulators, could have contributed to lower inflammation and patients’ mortality. The lack of bacterial or fungal co-infection data represents another limitation, as patients may have already presented bacterial co-infection at admittance which may have influenced the values of inflammatory biomarkers. A systematic review and meta−analysis with data from more than 30,000 patients showed a low prevalence of 4% of confirmed bacterial co-infection, while the use of antibiotics during the study period had been largely empirical (50). Lastly, the analysis of inflammatory biomarkers, while comprehensive, was limited by the availability of specific tests and the moment of admittance in relation to disease progression, which may not perfectly align across different waves and patient conditions.

This study has several notable strengths. First, it provides a comprehensive analysis of COVID-19 patients across multiple waves, in association with different VOC circulations, offering valuable insights into how the clinical and inflammatory profiles of the disease have evolved over time. The large sample size and inclusion of patients’ demographics, comorbidities, and vaccination status, and multivariate analysis adjusting for multiple confounders add depth to the analysis, enhancing the robustness and reliability of the findings. Additionally, the study’s detailed examination of a wide range of inflammatory and hematologic biomarkers allows for a nuanced understanding of the immune response associated with different COVID-19 variants. Furthermore, the use of standardized values for biomarkers facilitates comparisons across waves and enhances the clarity of trends observed. Overall, the study offers valuable real-world insights into the evolving clinical and inflammatory profile of COVID-19 in the context of emerging variants.

## 5 Conclusion

This study highlights the evolving landscape of COVID-19 through a comprehensive analysis of patient demographics, comorbidities, disease severity, and inflammatory biomarkers across different pandemic waves. The best inflammatory biomarkers for predicting severe/critical COVID-19 were CRP, dNLR, LDH, and NLR, while for predicting death outcomes, the best biomarkers were dNLR, NLR, LDH, and NPR. For all these biomarkers, the AUCs surpassed 0.8. In the multivariate analysis, the highest adjusted OR for death was described for dNLR, NLR, LDH, and NPR. Systemic inflammatory indexes are easy to use in clinical practice and accessible, allowing for the early identification of patients at high risk of severe evolution.

The Delta wave, characterized by the longest hospitalizations and the highest rates of severe cases and mortality, showed significant elevations in inflammatory biomarkers reflecting heightened systemic inflammation, while the Omicron wave had the lowest inflammatory status, suggesting a reduced severity of infection, even if we had the oldest patients during this wave.

The study, underscoring the dynamic nature of COVID-19 over time, brings a detailed analysis of biomarker trends that could provide valuable information for the early identification of patients at higher risk of severe outcomes, allowing for timely and targeted interventions.

## Data Availability

The raw data supporting the conclusions of this article will be made available by the authors, without undue reservation.
